# Hallucinogen-persisting Perception Disorder in a 21-year-old Man

**DOI:** 10.7759/cureus.4077

**Published:** 2019-02-14

**Authors:** Muhannad Kurtom, Ashley Henning, Eduardo D Espiridion

**Affiliations:** 1 Psychiatry, West Virginia School of Osteopathic Medicine, Lewisburg, USA; 2 Psychiatry, Frederick Memorial Hospital, Frederick, USA

**Keywords:** hallucinogen, persisting, perception, hppd, lsd

## Abstract

This is a case report of hallucinogen-persisting perception disorder in a 21-year-old man. Hallucinogen-persisting perception disorder, or acute hallucinogen-induced psychosis, is a rare disorder characterized by the presence of flashbacks of visual hallucinations as a result of previous hallucinogenic drug use. There is no standard of treatment, and management involves a combination of pharmaceuticals and lifestyle modifications. The combination of the rarity of the disorder along with the drastic impact this disorder has on a person's life makes this condition unique as compared to other substance-induced conditions.

## Introduction

Hallucinogen-persisting perception disorder (HPPD), or acute hallucinogen-induced psychosis, is the re-experiencing, when the individual is sober, of the perceptual disturbances that were experienced while the individual was intoxicated with the hallucinogen. It is a condition that causes significant distress or impairment in the social, occupational, or other important areas of a patient’s life, which is not attributable to hypnopompic hallucinations or any other medical conditions [1]. The pathogenesis for this disorder is unknown. However, the Diagnostic and Statistical Manual of Mental Disorders, Fifth Edition (DSM-5) classifies it as a substance-related and addictive disorder. The symptoms may manifest into any perceptual trepidation, but visual disturbances tend to predominate, with the most common being geometric hallucinations, flashes or intensifications in color, and false perceptions of movement [1]. There is no relationship between the occurrence of HPPD and the amount of substance used. The prevalence of this disorder is approximately 4.0% to 4.5% in people who have a history of hallucinogen use [1-2]. The most common comorbid conditions are panic disorder, alcohol use disorder, and major depressive disorder [1].

Although any hallucinogen can warrant the symptoms, the phenomenon of HPPD is primarily seen after LSD (lysergic acid diethylamide) use [3]. A distinguishing feature of HPPD is that reality testing remains intact (i.e., the individual is aware that the disturbance is linked to the effect of the drug). Schizophrenia, other substance-related effects, strokes, brain tumors, and head trauma should be ruled out before making an HPPD diagnosis.

## Case presentation

A 21-year-old African-American male presented to the emergency department at the local community hospital after an episode where the patient was reported to have flashbacks with hallucinations. He reported that the hallucinations were “tactile in nature” and that he could “still feel the cold breath” come out of him. Upon admission to the behavioral health unit, it was discovered that he took an unknown quantity of LSD seven months prior, and he claimed he was having flashbacks to that night, where he “blacked out” and then shortly after became aware of his surroundings to discover he was beating the man who had given him the LSD. There was, reportedly, “blood everywhere.”

Because of the altercation, the patient was arrested and is serving two years of probation with community service. The patient injured his hand at the time of the assault and is unable to work, so he is physically unable to fulfill the community service requirement. The patient is under significant distress because of the position he is in and reports that he does not want to go back to work as a lumberjack. Because he is currently unemployed, his stepfather has been threatening to evict him if he does not find a new job.

The patient admits he has symptoms of poor concentration, memory problems, sleeping difficulties, and is feeling isolated without a good support system. There are no prior psychiatric hospitalizations, and additional history was benign other than the incident he had seven months prior, after taking LSD.

The patient denied a history of suicidal thoughts and attempts. He denied alcohol use but admitted to marijuana abuse. He has never been married and has no children. Upon exam, the patient looked appropriate for his stated age. There was no psychomotor agitation or retardation. He maintained eye contact and spoke with coherence. The patient was sad and his affect was constricted. Immediate retention and recall, recent memory, remote memory, and fund of knowledge appeared to be fair. There were no referential or paranoid ideations. He denied any thought broadcasting, insertion, or withdrawal. He denied any delusions but admitted to visual hallucinations. He was paranoid and guarded. There were no suicidal or homicidal ideations, intents, or plans. He denied any problems with concentration. Insight and judgment were fair, but impulse control was poor by history. The patient was not taking any prescription, over-the-counter, or herbal medications.

On completion of the interview, the patient was observed in the behavioral health unit, and a decision was made to place the patient on clonazepam 1 mg four times a day. The patient fully recovered after four days, and he was then referred to a private local mental health center for follow-up.

## Discussion

Given the patient’s physical exam findings, it is likely the patient has hallucinogen-persisting perception disorder, as diagnosed by DSM-5 criteria. This case highlights the refractory nature of this patient’s delusions.

DSM-5 outlines three criteria for the diagnosis of HPPD. First, the patient presented with hallucinations following the cessation of the inducing substance, which, in this case, was LSD. Second, the patient is experiencing significant distress in the social and occupational aspects of his life. The third and final criteria for hallucinogen-persisting perception disorder states that the symptoms are not attributable to any other medical condition or disorder [1]. The patient’s past medical history and interview did not suggest any signs of medication side effects, schizophrenia, or head trauma. Brain imaging was negative, as expected, which allowed us to eliminate brain tumors, strokes, or other neurodegenerative disorders as a cause of his symptoms.

There is no mainstay of treatment for a patient carrying this diagnosis. Because anxiety can exacerbate symptoms, some studies have shown that group therapy and cognitive behavioral therapy (CBT) are effective ways to handle stress and establish a strong support network for those who are socially isolated [4]. Obviously, the treatment of HPPD should involve abstinence from all hallucinogenic substances and the treatment of the comorbidities mentioned earlier. Pharmacotherapy is very limited in resolving the neurological effects of hallucinogenic drugs. One observational study showed that all 16 subjects receiving benzodiazepines reported a reduced intensity and frequency of visual disturbances, and the improvement persisted during a six-month follow-up period [5]. Another study showed that high-potency benzodiazepines that have serotonergic properties may be more effective than low-potency benzodiazepines in the treatment of some patients with LSD-induced HPPD [6]. The patient, in this case, was given clonazepam, a high-potency benzodiazepine that facilitates gamma-aminobutyric acid (GABA) action by increasing the frequency of chloride channel opening, and his symptoms were relieved after four days [6-9].

The etiology of HPPD is unknown [3,5-6]. There are many theories that hypothesize the causal relationship between LSD and HPPD, but none of them have been validated. However, the long half-life and illicit nature of LSD use in uncontrolled environments may contribute to the frequency of flashbacks observed with this particular drug. Research looking at the long-term effects of hallucinogens estimates that flashbacks occur in roughly 25%-50% of individuals following LSD use [9]. Because these flashbacks caused significant distress in our patient, we were able to diagnose him with HPPD.

## Conclusions

For the reasons mentioned above, we believe this 21-year-old man has been experiencing hallucinogen-persisting perception disorder. This case highlights how perceptual disturbances after a single episode of hallucinogen use have long-term effects and represent real psychosocial distress that can be life-changing. We believe the patient received the best care possible, given his condition. The patient’s clonazepam regimen controlled his delusions. He is now in a freestanding mental health center where a patient-centered approach will be used for stress reduction and the maintenance of abstinence. By highlighting the devastating effects this disorder had on this patient, we hope to motivate more public awareness to decrease the incidence of hallucinogenic drug use. Due to the very low prevalence of this disorder, we believe it is valuable to document this case in order to motivate research to understand the etiology and pathophysiology of this phenomenon.
